# Supplementary material for: Genome-wide analysis reveals pathways important for the development and maturation of excitatory synaptic connections to GABAergic neurons
Source: G3 (Bethesda). 2026 Feb 8;16(4):jkag028. doi: 10.1093/g3journal/jkag028 (PMC13042287; doi:10.1093/g3journal/jkag028)
Supplement: jkag028_Supplementary_Data [file jkag028_supplementary_data.zip › File_S1_G3-2025-406255.docx]

**File S1.** Information on generated CRISPR strains

All CRISPR/Cas9 used in these studies were generated as described (Arribere et al., 2014).

***unc-63(uf198)***

Target sequences were selected on exon 2 of *unc-63*. Forward and reverse oligonucleotides were designed to contain the target sequence and overhangs compatible with *Bsa*I sites in plasmid Forward and reverse oligonucleotides were designed to contain the target sequence and overhangs compatible with *BsaI* sites in plasmid pPP13, a modified version of pRB1017 where the sgRNA scaffold was replaced with the sgRNA(F+E) scaffold (Ward, 2015). Forward and reverse oligonucleotides were annealed and ligated into pPP13 cut with *Bsa*I to create the gRNA plasmids. Plasmids were confirmed by sequencing with M13 reverse primer. A DNA mixture of pDD162 (P*eft-3*::Cas-9) (50 ng/µL), the gRNA plasmids (40 ng/µL each), pPP15 (50 ng/µL) (*dpy-10* target, a modified version of pJA58 were the sgRNA scaffold was replaced with the sgRNA(F+E) scaffold, the ssODN repair template for *dpy10(cn64)* (20 ng/µL) and the ssODN repair template for *unc-63* (Donor uf175) (80 ng/µL) was prepared in injection buffer and injected into N2 worms. Donor *uf175* contains a point mutation cCg/cTg resulting in a P/L amino acid change and a point mutation to eliminate an *AgeI* site for screening of mutants. Mutations in the *dpy-10* gene were used as a CRISPR co-conversion marker. The F1 progeny were screened for Rol and Dpy phenotypes 3-4 days after injection and then for the target mutation in the *unc-63* coding region by PCR and *AgeI* digest. The *unc-63(uf198)* mutant contains a cCg/cTg point mutation at codon 44 P/L.

***syd-2(uf193)***

Target sequences were selected on exon 18 of *syd-2*. Forward and reverse oligonucleotides were designed to contain the target sequence and overhangs compatible with *Bsa*I sites in plasmid pPP13, a modified version of pRB1017 where the sgRNA scaffold was replaced with the sgRNA(F+E) scaffold (Ward, 2015). Forward and reverse oligonucleotides were annealed and ligated into pPP13 cut with *Bsa*I to create the gRNA plasmids. Plasmids were confirmed by sequencing with M13 reverse primer. A DNA mixture of pDD162 (P*eft-3*::Cas-9) (50 ng/µL), the gRNA plasmids (50 ng/µL each), pPP15 (50 ng/µL) (*dpy-10* target, a modified version of pJA58 were the sgRNA scaffold was replaced with the sgRNA(F+E) scaffold, the ssODN repair template for *dpy10(cn64)* (20 ng/µL) and the ssODN repair template for *syd-2* (Donor uf174) (50 ng/µL) was prepared in injection buffer and injected into N2 worms. Donor *uf174* contains a point mutation Caa/Taa resulting in a Q/stop change and the generation if an *AflII* site for screening of mutants. Mutations in the *dpy-10* gene were used as a CRISPR co-conversion marker. The F1 progeny were screened for Rol and Dpy phenotypes 3-4 days after injection and then for the target mutation in the *syd-2* coding region by PCR and *AgeI* digest. The *syd-2(uf193)* mutant contains a point mutation CCA/TAA resulting in a Q/stop at codon 1091.
